# Ethics and Automated Systems in the Health Domain: Design and Submission of a Survey on Rehabilitation and Assistance Robotics to Collect Insiders’ Opinions and Perception

**DOI:** 10.3390/healthcare10050778

**Published:** 2022-04-22

**Authors:** Giovanni Morone, Antonia Pirrera, Paola Meli, Daniele Giansanti

**Affiliations:** 1 Dipartimento di Medicina Clinica, Sanità Pubblica, Scienze della Vita e dell’Ambiente, Università degli Studi dell’Aquila, 67100 L’Aquila, Italy; giovanni.morone@univaq.it; 2 Centro TISP, Istituto Superiore di Sanità, 00161 Roma, Italy; antonia.pirrera@iss.it (A.P.); paola.meli@iss.it (P.M.)

**Keywords:** ethics, robotics, social robot, rehabilitation

## Abstract

Background: The problem of the relationship between ethics and robotics is very broad, has important implications, and has two large areas of impact: the first is conduct in research, development, and use in general. The second is the implication of the programming of machine ethics. Purpose: Develop and administer a survey of professionals in the health domain collection of their positions on ethics in rehabilitation and assistance robotics. Methods: An electronic survey was designed using Microsoft Forms and submitted to 155 professionals in the health domain (age between 23 and 64 years; 78 males, mean age 43.7, minimum age 24, maximum age 64; 77 females, mean age 44.3, minimum age 23, maximum age 64) using social media. Results and discussion: The outcome returned: (a) the position on ethics training during university studies and in the world of work, (b) the organizational aspects hindered by ethics and those to be perfected in relation to ethics, (c) issues of ethical concern, (d) structured feedback on the usefulness of the methodology along with considerations of open text. Conclusions: An electronic survey methodology has allowed the structured collection of information on positions towards ethics in this sector. Encouraging feedback from the participants suggests the continuation of the study is beneficial. A continuation is expected, expanding the audience of professionals involved and perfecting the survey with the support of scientific companies.

## 1. Introduction

### 1.1. Robotics in Rehabilitation and Assistance

The Policy Department for Economic, Scientific and Quality of Life Policies of the European Parliament identified the most interesting applications of the care robots (CR)s [1]. The international body identified the following sectors:

1. Robotic surgery
2. Care and socially assistive robots
3. Rehabilitation systems
4. Training for health and care workers.

These sector are varied. Numbers two and three are both sectors connected to rehabilitation and assistance. Rehabilitation robotics are utilized in three areas [2]: (a) balance, (b) the lower limbs, and (c) the upper limbs. Two different technological solutions are used, based on exoskeleton technology and end-effector technology, with different implications in application [3]. Social robots are used in several multifaceted fields of the health domain for assistance and rehabilitation, including psychological, physical, and neurological rehabilitation [4].

### 1.2. Ethics and the Introduction of the Automated Systems in the Health Domain

The introduction of decision-making, therapeutic, and rehabilitation approaches, based on automatic systems, is radically changing the perspective of care in the health domain, raising important questions from an ethical point of view. As highlighted in [5], the use of automated systems in biomedical and clinical settings can disrupt the traditional doctor–patient relationship, which is based on the trust and transparency of medical advice and therapeutic decisions. An important criticism in [5] is that this approach, in which clinical decisions are no longer made solely by the physician, but to a significant extent by a machine using algorithms, decisions become non-transparent. They proposed a more ethical approach in which the decisions of these automatic systems are transparent, even to insiders. On the other hand, digital health is pushing towards an increasingly marked integration in the health domain, and access to healthcare records is more possible and easier than ever. All of this allows for the integration of automated systems and has the potential to transform medicine. To cite some examples: (a) Identifying previously unknown interventions that reduce the risk of adverse outcomes [6,7]. (b) Integration into medical decision workflows, from cellular and histological diagnostics [8] to functional and diagnostic imaging of organs [9], where scholars have highlighted how ethics is one of the most important challenges [10]. (c) Working in direct contact with patients through robots and other solutions based on artificial intelligence, where the implications, precisely for this reason, are broad and multifaceted [11]. Considering this, a conscious approach to ethics is now mandatory.

### 1.3. Ethics and Rehabilitation and Assistance Robotics

The issues of the ethics of automatic systems in the health domain are generally applicable to rehabilitation and assistance robotics. Rehabilitation and assistance robotics partially share ethical issues with automated systems; however, ethical issues in the latter field have also some peculiarities, such as direct work with patients [11]. The problem of the relationship between ethics and robotics is very broad and has important implications, ranging from integration of consent to cybersecurity [12].

Ethics in the field of rehabilitation and assistance robotics has two large areas of impact to many issues, which are shared with automatic systems in general. The first large dimension is research conduct, development, and use in general [13,14]. This concerns both social robots and robots in used in rehabilitation [15,16]. The second large dimension concerns only social robots and is the design of machine ethics [17]. 

Regarding the first dimension, Stahl and Coeckelbergh [13] identified, as a first important topic, the technological impact to daily life and the health domain. The implications of replacing humans with machines in the health domain must be addressed [18,19,20,21,22,23,24,25]. Therefore, the following issues are of particular importance:

- the implications to work of those in contact with the patient;
- consequently, the quality of care in relation to what has been defined as a risk of dehumanization or even cold care.

When focusing on the replacement, of humans it is necessary to consider:

- the implications of the decision-making autonomy of the decision-making robot (e.g., margins and impact);
- the chain of responsibility for the decision-making robot;
- the risk of deception, such as the risk of creating false friendships with social robots;
- the trust in placing a patient (for example a frail person) in the hands of a robot.

Then there is a second important topic, connected with the cybersecurity applied to the mechatronic. The following issues are important:

- privacy and data protection;
- safety and avoidance of harm.

Gordon highlighted a second large dimension [17], which addresses the problems of ethics when implementing ethical rules in a moral robot. All this is important to broad and interdisciplinary sectors, such as artificial intelligence. The criticality of this sector is given by the fact that anyone who programs ethics in a computer must have specific training on ethics [26,27,28,29].

Etemad-Sajadi et al. [30] also categorized (after a review of the state of the art) six specific strategic items of concern related to ethics in this sector, which must be taken into consideration in studies on the integration of consent: social cues [21,22,28,29], trust and safety [13,30], autonomy [13,30,31], replacement [30,32,33,34], responsibility [13,30,34], and privacy and data protection [13,30,35]. 

Robotics in this sector have also developed a strong integration with virtual reality [3] and is moving towards an important integration with artificial intelligence [36]. When we focus on ethics, we must even consider pursuits such as these.

Furthermore, we must not forget an emerging ethical issue in automatic systems in general which is also applicable here: the implication of equity [37]. Health systems rely on commercial prediction algorithms to identify and help patients with complex health needs. In [37], it was shown that a widely used algorithm in automated systems, affecting millions of patients, exhibited significant racial bias. This must be particularly considered and avoided even in robotics, which has already been used in the health domain as a niche rehabilitation and assistance system.

### 1.4. Hypothesis of the Study

Ethics is assuming an essential and important role in the introduction of rehabilitation and assistance robotics in the health domain. It is therefore crucial to consider the ethics in studies on the integration of consensus.

Professionals who are involved in patient interaction will increasingly play a key role in interacting with robotics in a wide range of activities, ranging from the execution of robotics-based protocols to application programming in robots. The ethics of the integration of rehabilitation and assistance robotics is passed through the opinions and consent of these professionals.

We hypothesized that it was possible to focus on these figures and to remotely administer, through the mobile technology, an electronic survey to collect demographic data and to collect information on professionals’ training and their relationships with ethics. 

### 1.5. Objectives of the Study

- Develop and administer a remote electronic survey that would allow: (a) the collection of demographic data and (b) the collection of data on the training on ethics and the self-perception of the impact of the ethics, concerns, and suggestions.
- To collect feedback on the investigation and opinions on this topic.

## 2. Methods

### 2.1. Participants and Procedure

#### 2.1.1. The Selected Tool and the Adequacy of Regulations

This questionnaire project was previously carefully discussed with experts on data protection. It complies to regulations (national and international) of the European GDPR 679/2016 and the Italian Decree 101/2018. The questionnaire was anonymous, and the topic did not concern clinical trials on humans or animals. Furthermore, it did not involve participants with pathologies. In consideration of this, after a pre-check, the approval of an ethics committee was not deemed necessary (which would have required a long time, incompatible with this study). However, to improve the privacy aspects, we did not proceed via e-mail and to avoid requesting the municipality of residence (in small municipalities, this it could lead to identification). In this study, the software Microsoft Forms was chosen. Our company has this tool centrally installed. Users have this tool, among other applications, on the Microsoft 365 App Business Premium suite (the maximum limit of participants/submission is 50,000). All users, internal and external, can access through their own domain account guaranteed by corporate cybersecurity standards (which are forced to comply with international regulations) to develop surveys by Forms. The developed products, shared with external subjects, are supported in each phase both by the system security tools/system policy and network security, managed by the company firewall, which can also perform specific checks on the IPs (registering, for example, duplicate access for further data processing). The data acquired through a survey developed by means of Microsoft Forms, are protected by corporate security systems. In fact, they are a register for legal purposes in case of the presence of sensitive data (for which the creator of the survey is responsible) and are protected by corporate cyber security systems, guaranteeing (at least from the system point of view) the inviolability of the data and the maintenance, according to article five of the GDPR 679/2016, for a period of time not exceeding the achievement of the purposes for which they are processed. Microsoft Forms is the tool recommended by the company’s Data Protection Office. A choice different than Forms would have required a specific report and cybersecurity study; therefore, the authorization to use it would not have been guaranteed. The use of both an internal recommended tool (respecting the cybersecurity) and the choice to submit the survey anonymously (without requiring sensitive data) simplified the process of launch, that, after a preliminary check, did not need specific authorizations. 

#### 2.1.2. Main Characteristics of the Chosen Tool

The chosen electronic survey (Microsoft Forms), based on the above considerations, allows submission via an internet link accessible in a secure manner, reported above by means of an https link. This tool allows sending via multimedia systems, chats, emails, webs, social networks, in a simple way, and data collection automatically. It avoids all laborious paper submission activities as well as laborious data collection, not free from errors, due to transcription from paper to an electronic database for processing.

Once sent by the administrator, the electronic survey is opened by the receiver, filled in, and by means of a simple sending confirmation, allows the data to be uploaded in real time into a database. At any time, and in particular at the fixed and scheduled deadline for sending replies, data are accessible both in the form of post-processing reports and in Microsoft Excel for other statistical post-processing analyses.

**Figure 1:**
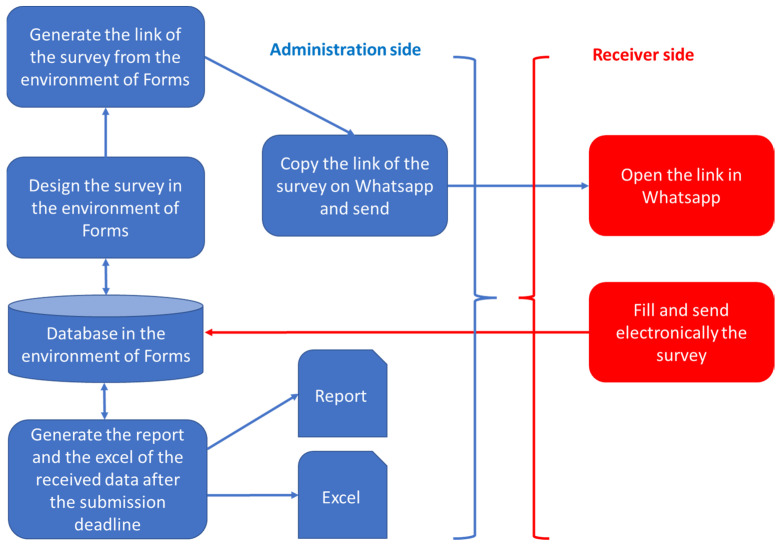
An example of the submission flow based on a possible applicable tool (WhatsApp).

An example of this flow is shown in Figure 1, where the submission is illustrated using one of the possible tools (WhatsApp).

A subtitle can also be inserted on each question that guides the compiler giving greater certainty and security on what to insert. In [38,39], where there are links and pdf printouts of the survey (described in the following), you can see, for example: (a) A subtitle of the heading, before the questions of the survey reports, “The survey is dedicated to the healthcare professionals. It is anonymous. The submitted data are protected by cybersecurity.-PLEASE help those who are not technology experts to fill in the questionnaire-” giving information and clarifications (also on security aspects). (b) In the first question, a subtitle gives clarifications and information on the security to the participants. (c) A subtitle helps the participants in the second question. 

#### 2.1.3. The Tool: Structure 

The tool included four sections: (a) A section dedicated to the information of the participants, asking for consent to the survey, information related to demographic data (sex, age), and a brief curriculum. (b) A section with graded questions and Likert scales [40] dedicated to self-perception of the training and impact of ethics in, e.g., concerns and suggestions in the workplace. (c) A section with graded and open questions asking for opinions on the methodology. 

The original survey is in Italian and is closed and no longer accessible.

We have translated a version from Italian into English for editorial purposes. A link to the interactive tool is available online at [38].

The link to the pdf printout is available online at [39].

#### 2.1.4. Submission and Participants

**Figure 2:**
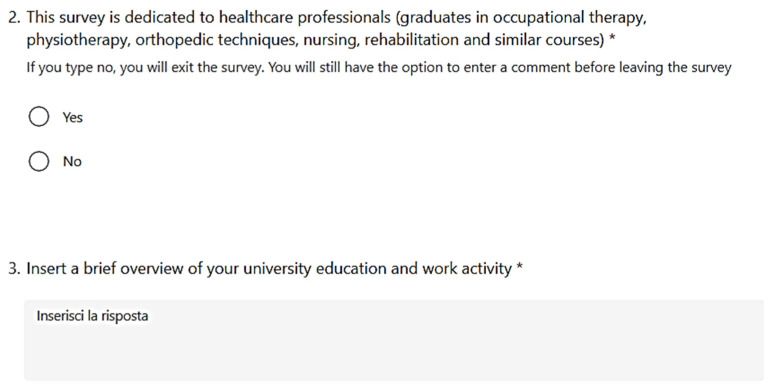
The section of the questionnaire dedicated to inclusion in the study.

The only prerequisite that we set ourselves, to limit the articulations of the study, was to focus on healthcare professionals (graduates in occupational therapy, physiotherapy, orthopaedic techniques, nursing, rehabilitation, and similar courses). Based on a dedicated section, respondents were included (or excluded) according to compliance (or non-compliance) with these requirements (Figure 2).

The electronic survey was sent on 15 October 2021. The tool remained active until 15 December 2021. The submission took place through social media, such as Facebook, LinkedIn, Twitter, Instagram, WhatsApp, association sites or scientific societies, and in general, a peer-to-peer dissemination.

We have also encouraged both the spread of the electronic survey and the support in filling out for those who are less familiar with digital technology (also strongly specifying this in the electronic survey introduction).

Table 1 shows the demographic characteristics of the participants in the study.

### 2.2. Measures

The survey considered various parameters for the collection and evaluation of information, some of which were used in this work and others will be explored further later. The following parameters were considered to be related to the submission rate: the total submissions, the total number of people who opened the survey but didn’t participate, the total number of those who could not be included, and the total number of people.

In the survey there are also graded questions and open questions (for comments) to have feedback on the administration process. We established a six-level psychometric scale for the graded questions. The assignable values ranged from a minimum score = 1 to a maximum score = 6. Considering this, a theoretical average value (TMV) can be identified as:

(1) $$TMV=\frac{1+6\;}{2}=3.5$$

The value in Equation (1) is equally distant, with an absolute value of 2.5 both from the maximum assignable score = 6 and the minimum assignable score = 1.

It is therefore possible to assign a minimum score of one and a maximum of six with a theoretical mean value (TMV) of 3.5. We can refer to the TMV for comparison in the analysis of the answers. An average value of the answers below the TMV indicates a more negative than positive response. An average value above the TMV indicates a more positive than negative response. The outcome of the open questions was investigated qualitatively. The best five were selected on the basis of a ranking formed on the basis of an evaluation that took into account on impact and significance.

**Figure 3:**
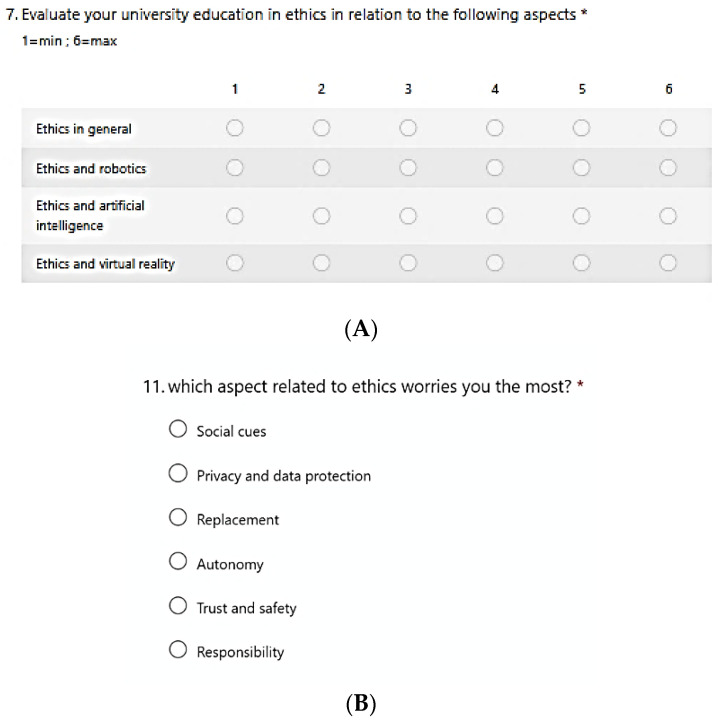
(**A**) Survey section (Likert’s scale) dedicated to questions on academic training. (**B**) The choice question with ethical aspects of concern.

In the survey, there are also Likert scale questions (as for example the question 7, Figure 3A) and choice questions (Figure 3B). We established a six-level psychometric scale for the Likert scale questions as the graded questions. 

### 2.3. Statistics

We used the Smirnov–Kolmogorov test for testing the normality, as it is preferable for large samples such as ours. We applied the χ^2^ test (with a *p* < 0.01 for the assessment of the significance) in the frequency analysis. 

We applied the Student’s *t*-test (with a *p* < 0.01 for the assessment of the significance) when investigating the difference between the parameters.

The Cohen’s d effect size was estimated for the assessment of the adequacy of the sample. Furthermore, the Cronbach’s α value was assessed for the psychometric sections of the electronic survey. The software SPSS version 24 was used in the study. 

## 3. Results

The results are organized into four paragraphs. The first paragraph reports the results of the administration. The second paragraph reports a statistical analysis of significance, firstly, the statistical tests then applied to the analysis of the outcome. The third paragraph is dedicated to the central analysis of the study, i.e., the analysis of the participants’ opinion/perception. The fourth paragraph reports the analysis of the participants’ feedback on the method.

### 3.1. Submission 

The electronic survey was sent on 15 October 2021. The tool remained active until October 25. A total of 91.74% of responses were obtained in the first four days. We sent 202 electronic surveys and 14 subjects did not give the consent. A total of 33 subjects could not be included because they did not pass the inclusion process reported in Figure 2. A total of 155 participants were included, as can be observed in Table 1.

### 3.2. Preliminary Test of Statistical Significance

Preliminarily to the analysis, we applied the selected tests to verify the normality of the data, the adequacy of the sample, and the sensitivity of individual factors.

We tested the distribution of age for the sample with the Smirnov–Kolmogorov test of normality, which is suitable for large samples such as ours. The null hypothesis was that our data followed a normal distribution. We achieved *p* = 0.53. Because *p* > 0.05, we accepted the null hypothesis. We were therefore working with a normal distribution.

The Cohen’s d effect size was 0.499, indicating that the proposed sample was suitable (N > 60). Furthermore, the Cronbach’s α value of individual factors was assessed for the graded questions and the Likert questions. It reported a value = 0.8, i.e., a good level of reliability.

### 3.3. The Ethics Perception on the Insiders 

You can refer to [38,39] for the questions in detail.

Question no. 6, related to robotics training, reported a score of 3.61, just above the TMV. The Likert scale in question 7 (Table 2), relating to the evaluation of training on ethical aspects, reported an evaluation lower than the TMV for all modules (ethics in general, ethics and robotics, ethics and artificial intelligence, and ethics and virtual reality).

The same Likert scale applied to the current knowledge on the job (Table 3), with question number 8 [38,39], reports a better situation (above TMV for all modules), presumably thanks to the improvements in knowledge triggered in the workplace, due to a greater sensitivity determined on the issue by professional associations or scientific societies.The Student’s *t*-test was applied to verify the significance of the difference between the same modules in the two Likert scales. The test of the four applications always reported a high significance of the difference (*p* < 0.01).

In regards the two Likert questions:

9—“Regarding the organizational aspects, what would you suggest to improve in terms of training and/or in-depth study in the field of ethics in robotic rehabilitation in the neurological field?” and

10—“Regarding the organizational aspects, do you think that ethical issues can hinder ?” 

We have decided to report the details of the answers together with the average values. In this manner, we can compare the frequencies of positive answers (more positive than negative: values of 4, 5, and 6) with the frequencies of the negative answers (more negative than positive: values of 1, 2, and 3).

Both Likert questions reported a higher frequency of positive responses (more positive than negative: values of 4, 5, and 6). The χ^2^ applied to each of the elements of the two Likert questions always showed a high statistical significance (*p* < 0.01). 

As for the Likert scale in Table 4 (question 9), all of the elements proposed showed a need for improvement in terms of training and/or in-depth study. Among the elements proposed (the relationship with the robotic devices, the impact of virtual reality, the use of social robots, the use of artificial intelligence, the integration between the artificial intelligence and virtual reality with the robotics, regulation issues) the element that showed the highest need for intervention was “the regulation issues”.

As for the Likert scale in Table 5 (question 10), all of the elements proposed showed that ethics was always considered a hindering issue. Among the elements proposed (the use of the robotics in general, the integration of robotics with artificial intelligence, the integration of robotics with virtual reality, the use of the social robot), the element that showed the greatest criticality regarding ethical aspects was “the use of the social robot”.

Table 6 reports the output of the choice question related to the question “which aspect related to ethics worries you the most?” connected to the strategic items identified in [20].

The suggestion that had more answers was the replacement, with a percentage equal to 71.61%. Data security was viewed with less concern than all the other proposed aspects. The χ^2^ applied to the frequencies of the choices showed a high significance (*p* < 0.01).

### 3.4. Feedback from the Participants

**Figure 4:**
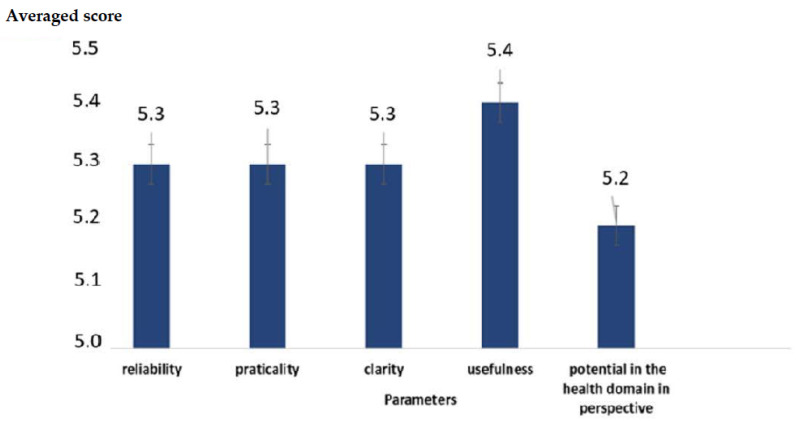
Averaged score for the different parameters proposed to assess the opinion of the participants.

We analyzed the feedback obtained through open and graded questions. Figure 4 shows the high averaged values (score > 5) of the answers to the graded questions, highlighting a high degree of acceptance of the methodology in regard to all of the proposed parameters: reliability, practicality, clarity, usefulness, and potential. Furthermore, the Cronbach’s α value of individual factors was assessed. It reported a score = 0.73, i.e., an encouraging level of reliability.

The best six were selected based on a ranking considering both impact and significance.

They are reported in Box 1. The selection highlights: (a) a desire to further investigate ethical aspects and (b) concern about the impact of some of them.

Box 1 **Selected open answers.**

                              **Comment**

*I am convinced that ethical aspects are given little space both to university courses and in the workplace. This applies to the entire healthcare sector and also to robotics*

*I think the introduction of social robots is rapidly approaching the era of robots with its own ethics. The problem is that if something is wrong in the design, there is a risk not only of malfunctions, but an impact on the patient’s physical and mental health.*

*I fear that the introduction of robotics risks leading to a dehumanization of medical care and the ethical impacts are considerable. I am distinctly against it*

*I think that with the introduction of robotics in the medical field, the impact of ethics on the various professions will have to be seriously assessed, and consequently the ethical codes of the various professional orders will have to be heavily revised.*

*I think that in the future it will be necessary to work on many rehabilitation protocols and readjust them to the use of robots after heavy analysis of the impact of ethics.*

*I think that one of the critical aspect that we must consider is the equity in providing the robotic care. It should be avoided the discrimination of the less well-off*

## 4. Discussion

Ethics have an important impact on the use of robotics in rehabilitation and assistance. [14,15] The implications are considerable and concern all aspects related to human replacement, data management and, as in the case of social robots, the programming of ethics itself. Regarding ethics programming, some studies have shown that programmers lack adequate cultural bases [17], which can create significant problems, including cybersecurity [12]. Regarding ethics in research and development, many studies, not all focused on the health domain, have identified some elements of concern (social cues, privacy and data protection, replacement, autonomy, trust and safety, and responsibility) [13,30]. In [30], it was highlighted how a population survey showed that the most critical was replacement.

**Figure 5:**
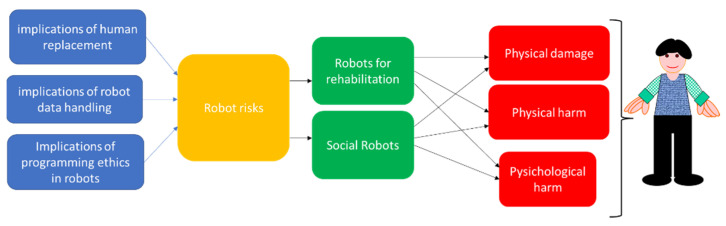
Model of impact of ethical issues.

In general, by adapting the model proposed in [12], it can be highlighted how the ethical issues can have a direct impact on both physical and psychological health (Figure 5).

The impact on both physical and mental health and on IT security makes it necessary to pay particular attention to ethics. This attention must start from the training processes during studies and continue in the workplace. Both the perceived level of knowledge in this area and the insiders’ opinions need to be monitored. Recently, in line with our position, many studies are analysing the ethical problems in many areas of robotics [41,42] including the one we have taken into consideration [43].

Our study, focused on the health domain, proposed a survey on insiders with two polarities. The first consists of a point of view that deals with robotics and related technologies: (a) the training position during university studies and in the world of work, (b) the organizational aspects hindered by ethics and to be perfected in relation to ethics, and (c) aspects of ethics of concern. The second point of view consists of both structured and open-ended feedback on the proposed methodology. This study highlights an increase in mastery of ethical aspects in the workplace, a criticality in the regulatory aspects related to ethics and a major obstacle in social robotics. In line with the study conducted in [30], one of the aspects of greatest concern was considered to be the replacement of humans. We must consider that the study conducted in [30] was not focused on the health domain, but instead on service applications of robotics. Furthermore, ordinary citizens were included in this study, while in our study, insiders from the health domain were included who also expressed their perceptions on other issues such as training, organization, and needs for further study and obstacle. They also reported the positive feedback of high acceptance of the methodology with impressions in an open text form.

Our study is in line with other approaches in this field considering training [44]. The study reported in [44] considered the ethics in training, where despite a broad consensus on the ethical dimensions of the teaching profession, little is known about how teacher candidates are being prepared to face the ethical challenges of contemporary teaching. It presented the results of an international survey on ethics content and curriculum in initial teacher education involving five Organization for Economic Co-Operation and Development countries—the United States, England, Canada, Australia, and the Netherlands. Our study, as in [44], reiterates both the importance of training in ethics and the importance of surveys in these studies. Furthermore, our study provides, in a certain sense, a complementary result. While the study reported in [44] is focused on the role of trainers, our study is focused on those who participate as a learner in training courses in a sector where ethics has a particular impact.

Our study is also in line with what is highlighted in [45], where the importance of ethical aspects was addressed; it was highlighted that ethics is among the key aspects to be considered in the integration of robotics consensus (guidelines, health technology assessment, and consensus conferences). Indeed, at the Italian national Conference of consent in this field, through the activity of a dedicated working group [46], the following were highlighted: the important role ethics plays and specific recommendations on this issue, such as a stimulus for stakeholders and researchers, which we followed with the launch of this study. The state of the perception of ethics is as an obstacle on the part of insiders; this is in line with the results of the Likert questions reported in Table 5. Considering the above, the general added value of our study consists of a methodology, based on an electronic survey to investigate this issue, adapted to a category of insiders involved in interaction in the health domain.

From a more specific point of view, the study returns the following three added values:

The first added value is the electronic survey product which, although in a prototypal form, can also be investigated and used in future applications as a monitoring tool and by scientific societies.

The second added value is represented by the first outcome of quantitative and indicative data from the study.

The third added value is represented by both structured feedback and observations from patients.

### Limitations

The first two limitations are those typical of the electronic surveys, as those ones indicated in national studies based on these tools [47], i.e., the willingness and the type of administration, that includes the participants in the study with a “fishing on the pile procedure”, that we strained to compensate for by designing and applying a robust statistic.

A third limitation is that the survey is a prototype. It can be improved through the intervention of scientific societies to include other professionals, and then used in consensus integration initiatives, such as in consensus conferences [45,46].

## 5. Conclusions

Ethics represents a key aspect for the introduction of robotics in the world of rehabilitation and assistance. The study focused on the category of health domain insiders. A targeted survey was developed on this issue. This survey made it possible to obtain various outcomes. First, the study reported both the state of academic training on this topic and the knowledge subsequently integrated into the world of work. Second, the study highlighted, in terms of perceptions, strong criticalities of the regulatory aspects regarding organizational aspects and strong ethical obstacles on the introduction of social robots. Third, among the aspects of concern the most relevant was the replacement of humans with robots. The study also reported a high acceptance of participants and suggests future developments in these areas in collaboration with scientific societies.

## Figures and Tables

**Table 1:** Demographics characteristics.

Participants	Age and Gender
155 professionals of the health domain with specific academic training	Age between 23 and 64 years. 78 males (mean age 43.7; minimum age 24, maximum age 64) 77 females (mean age 44.3; minimum age 23, maximum age 64)

**Table 2:** Output from the Likert scale “Evaluate your university education in ethics in relation to the following aspects”.

Question	Score
Ethics in general	3.04
Ethics and robotics	2.87
Ethics and artificial intelligence	2.93
Ethics and virtual reality	2.99

**Table 3:** Output from the Likert scale “Evaluate your current knowledge in ethics in relation to the following aspects”.

Question	Score
Ethics in general	3.64
Ethics and robotics	3.51
Ethics and artificial intelligence	3.52
Ethics and virtual reality	3.53

**Table 4:** Output from the Likert question “Regarding the organizational aspects, what would you suggest to improve in terms of training and/or in-depth study in the field of ethics in robotic rehabilitation in the neurological field?”.

Question	N(1)	N(2)	N(3)	N(4)	N(5)	N(6)	Score
The relationships with the robotic devices	2	6	4	27	40	76	5.01
The impact of the virtual reality	1	6	15	18	62	53	4.89
The use of social robots	3	7	16	18	64	47	4.77
The use of Artificial Intelligence	3	7	17	18	70	40	4.71
The integration between the artificial intelligence and virtual reality with the robotcs	4	5	14	18	68	46	4.80
The regulation issues	0	0	1	5	64	85	5.50

**Table 5:** Output from the Likert question “Regarding the organizational aspects, do you think that ethical issues can hinder”.

Question	N(1)	N(2)	N(3)	N(4)	N(5)	N(6)	Score
The use of the robotics in general	2	5	5	37	30	76	5.04
The integration of robotics with artificial intelligence	2	7	13	18	62	53	4.87
The integration of robotics with the virtual reality	4	6	15	19	54	57	4.83
The use of the social robot	0	0	1	11	11	132	5.77

**Table 6:** Output from the choice question “Which aspect related to ethics in Robotics worries you the most?”.

Question	Number of Choices
Social cues	4
Privacy and data protection	2
Replacement	111
Autonomy	13
Trust and safety	5
Responsibility	10

## Data Availability

Not applicable.
